# Supplementary material for: Co-axial heterostructures integrating palladium/titanium dioxide with carbon nanotubes for efficient electrocatalytic hydrogen evolution
Source: Nat Commun. 2016 Dec 12;7:13549. doi: 10.1038/ncomms13549 (PMC5159813; doi:10.1038/ncomms13549)
Supplement: Supplementary Information — Supplementary Figures 1-22, Supplementary Tables 1-2, Supplementary Notes 1-4 and Supplementary References [file ncomms13549-s1.pdf]

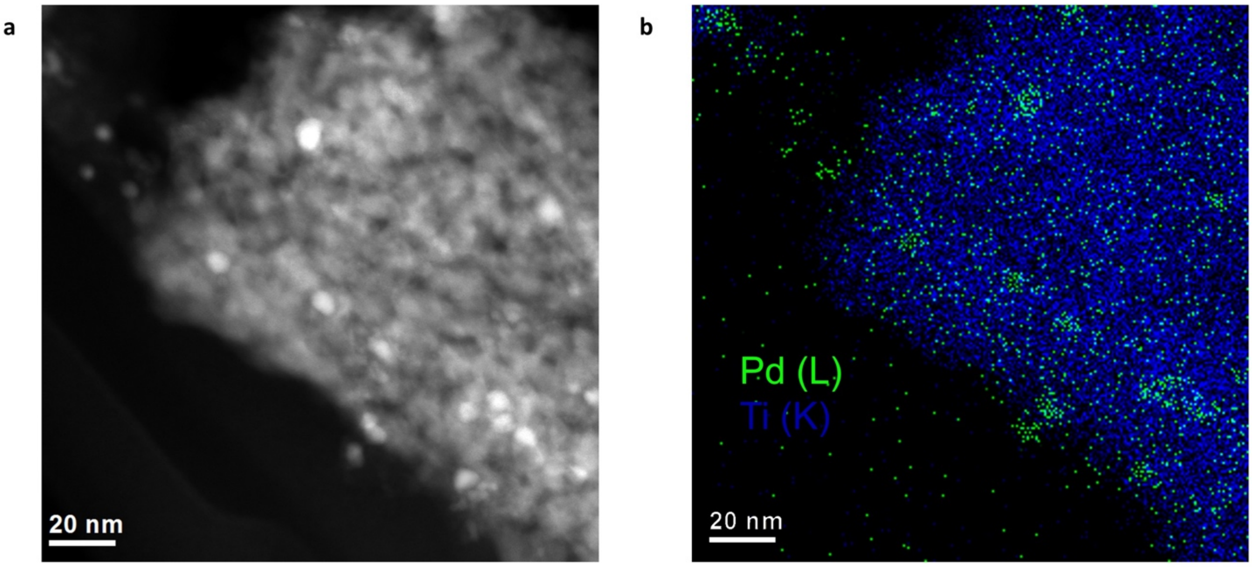

Supplementary Figure 1. HRTEM and STEM-HAADF images of f-MWNCTs@Pd/TiO_2. STEM-HAADF image (**a**) and the corresponding EDXS map (**b**) for a **f-MWCNTs@Pd/TiO_2** wire.

Pd nanoparticles were identified in the nanocomposite thanks to the High Angle Annular Dark Field (HAADF) STEM images and tomography. Supplementary Fig. 1 shows the STEM-HAADF (Z contrast) image and the corresponding Energy Dispersive X-Ray Spectroscopy (EDXS) for the **f-MWCNTs@Pd/TiO_2**. Here the naked carbon nanotube is in the upper left corner of the picture, while Pd nanoparticles can be distinguished from the TiO_2 structures due to their higher atomic number and thus higher brightness. The STEM-EDXS maps can be used to confirm this result. Supplementary Fig. 1b shows the elemental distribution of Ti-K (blue) and Pd-L (green) taken on the same area of Supplementary Fig. 1a, demonstrating that the brighter particles correspond to Pd nanoparticles.

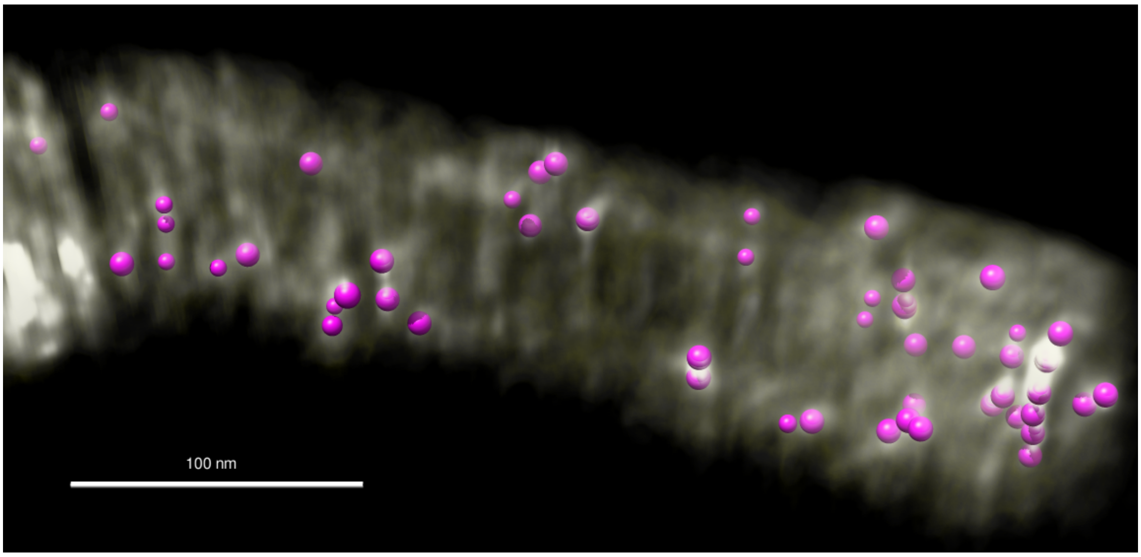

Supplementary Figure 2. 3D STEM tomography. 3D rendering of a **f-MWCNTs@Pd/TiO_2** wire after tomographic reconstruction as for Fig. 1b in the manuscript. At the regions with high densities (corresponding to the Pd particles) we placed spherical markers (yellow spheres in Fig. 1b, here in violet for better contrast), using the a-priori knowledge of approximate spherical shape and dimension of the Pd particles. We left the bottom left portion of the wire with no markers for better visualization of the raw density map.

A 3D tomography reconstruction of a **f-MWCNTs@Pd/TiO_2** wire was obtained from HAAD-STEM projections and is presented again in Supplementary Fig. 2 for clarity. The reconstruction was obtained using the TomoJ plugin,[4] by a weighted back projection (WPB) followed by 20 SIRT iterations to reach convergence in the reconstruction. The 3D rendering (isosurfaces and spherical markers) was done using the UCSF Chimera package.[5]

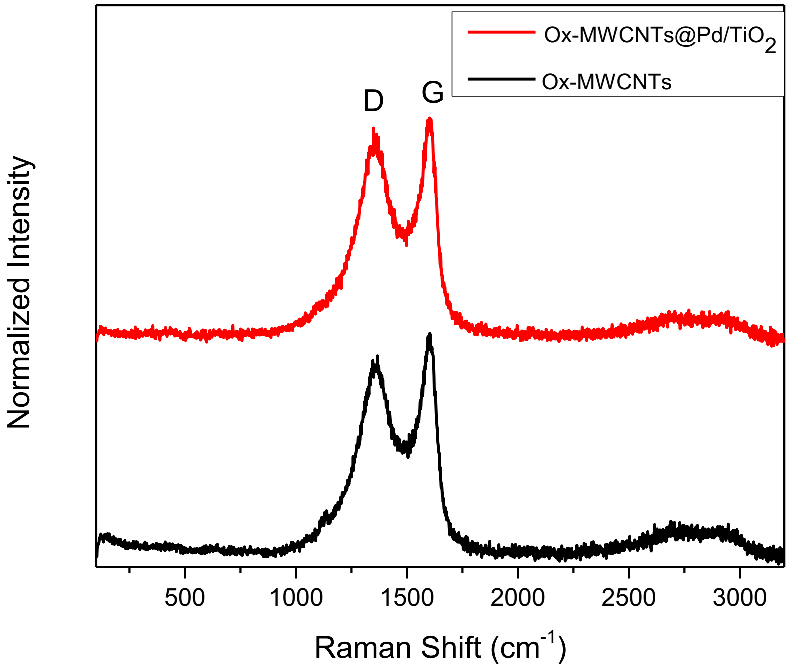

Supplementary Figure 3. Raman spectra of the nanohybrids. Raman spectra of **Ox-MWCNTs** (black) and **Ox-MWCNTs@Pd/TiO_2** (red).

In Supplementary Fig. 3 it is shown a comparison of the Raman spectra between **Ox-MWCNTs** and **Ox-MWCNTs@Pd/TiO_2**. The characteristic peaks of the graphitic framework of the **MWCNTs** are observed with the defect-induced D band at ~1350 cm^{-1} and the breathing mode of the polyaromatic C=C bonds evidenced by the G band at ~1600 cm^{-1}. After hybridization with the **Pd/TiO_2** inorganic phase, we observed no signature of any specific TiO_2 crystal phase, confirming the amorphous nature of titania.

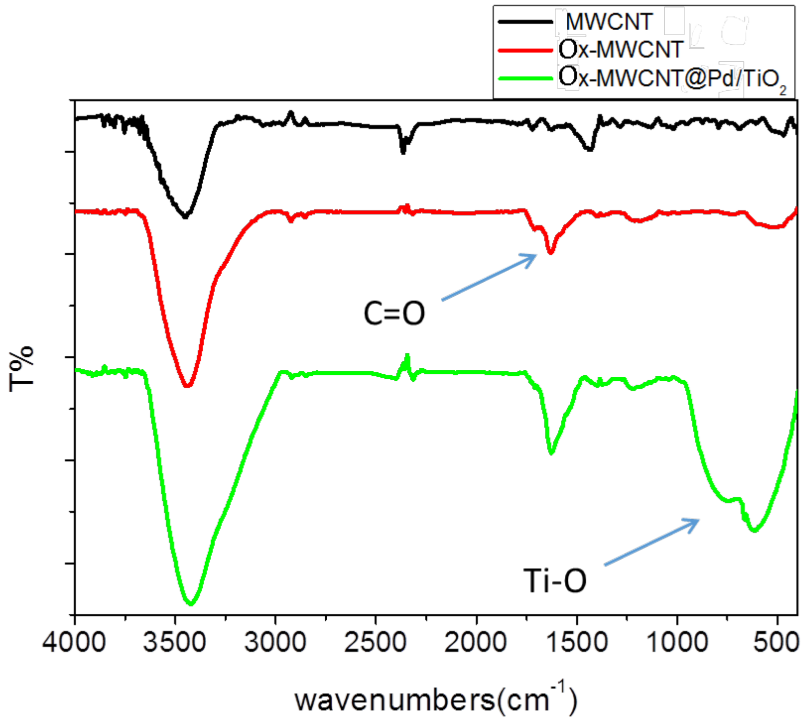

Supplementary Figure 4. FTIR spectra of the nanahybrids. Pristine MWCNTs (**MWCNTs**, black), **Ox-MWCNTs** (red) and **Ox-MWCNTs@Pd/TiO_2** (green).

In the FTIR spectrum of Supplementary Fig. 4, we observed a clear change in the spectrum by passing from pristine **MWCNTs** to **Ox-MWCNTs** to **Ox-MWCNTs@Pd/TiO_2**. Oxidation reaction of the nanotubes introduces COOH groups, visible with the C=O stretching at 1716-1625 cm^{-1} and small OH bending at 1403-1356 cm^{-1} (the OH stretching is overlapping with water/moisture signals). After hybridization with Pd/TiO_2, the Ti-O-Ti stretching modes became evident (762-600 cm^{-1}).

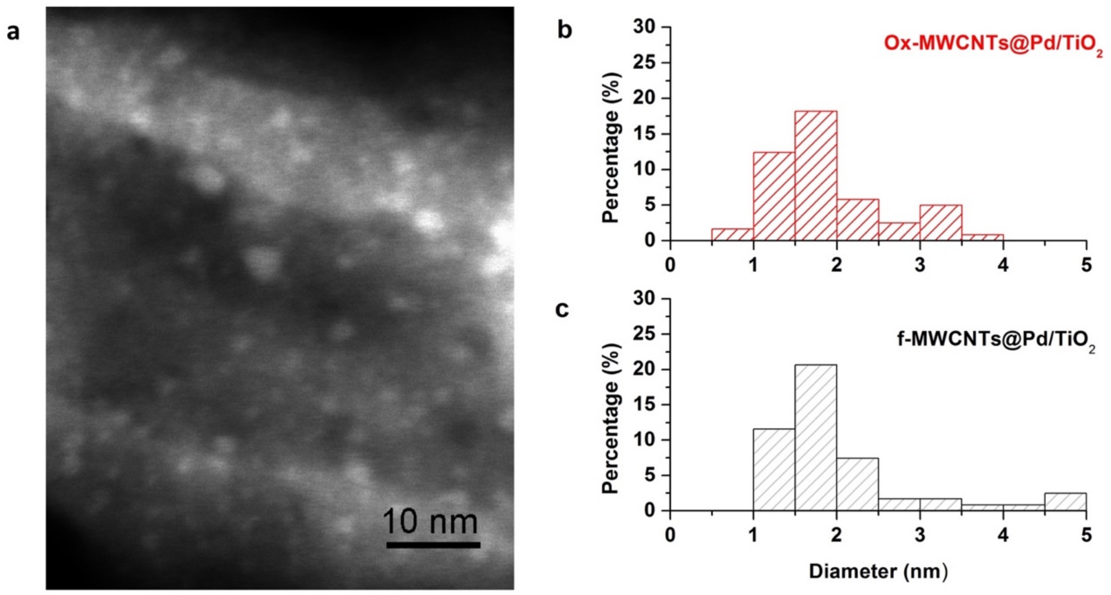

Supplementary Figure 5. Effects of calcination on particles size. (**a**) STEM-HAADF image of the **f-MWCNTs@Pd/TiO_2** nanocomposite. (**b,c**) Particle size analysis of the two materials, **Ox-MWCNTs@Pd/TiO_2** before calcination (**b**), and **f-MWCNTs@Pd/TiO_2** after calcination (**c**).

To highlight possible effects of the thermal treatment on the morphology of the hierarchical nanostructures, we performed particles size analysis focusing on the behaviour of the Pd catalytic cores. ECSA measurements (Electrochemical Surface Area) of the fresh and calcined nanohybrids before and after calcination are herein reported. ECSA analysis of the PdO peak,[1-3] reveals that the thermal treatment does not affect significantly the Pd accessibility, with a slight increase in Pd electroactive area following calcination. ECSA values of 0.0058 cm^2 and 0.0065 cm^2 were obtained, before and after thermal annealing at 350°C, respectively. In agreement with ECSA, the Pd NPs size analysis confirms the very small size of the nanoparticles, which is not altered by the calcination treatment (Supplementary Fig. 5).

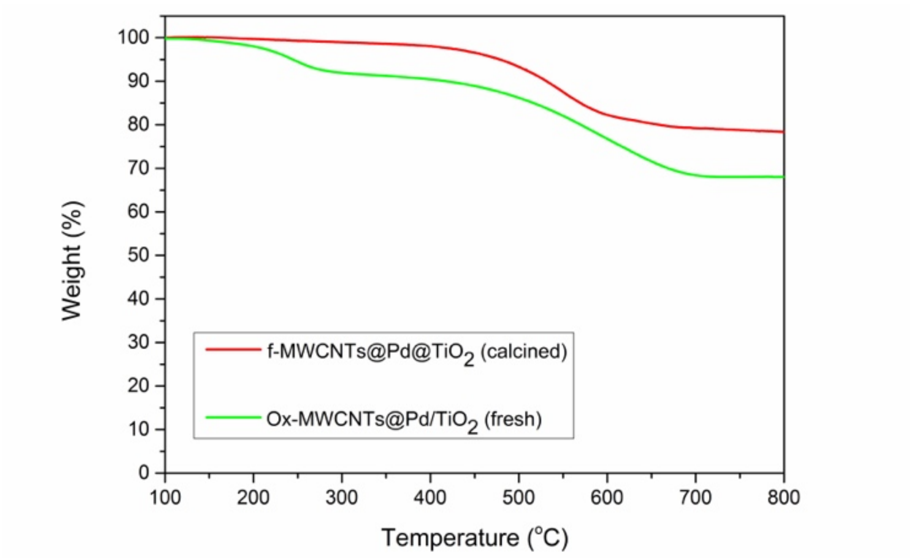

Supplementary Figure 6. Effects of calcination on TGA. TGA comparison of the two materials, fresh (**Ox-MWCNTs@Pd/TiO_2**) and calcined (**f-MWCNTs@Pd/TiO_2**). It is evident how upon calcination, the material does not present anymore the weight loss due to the organics.

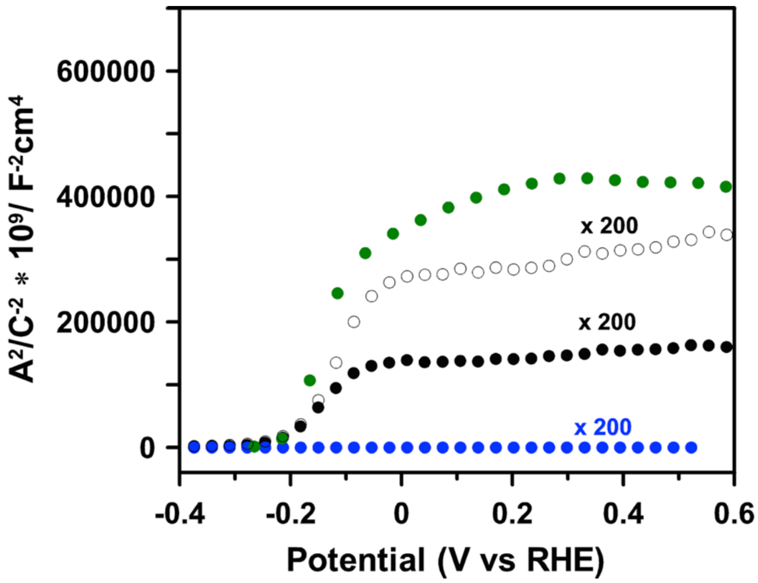

Supplementary Figure 7. Mott-Schottky analysis of the nanohybrids. Mott-Schottky (C^{-2} *vs.* applied potential) plots for the various nanostructured systems in phosphate buffer, pH = 7.4, obtained at a fixed frequency of 1 kHz: **Ox-MWCNTs(20%)@Pd/TiO$_2$** (●), **Ox-MWCNTs(15%)@Pd/TiO$_2$** (○), **Pd/TiO$_2$** (●) and the pristine electrode surface (●).

If we compared the **Pd/TiO$_2$** nanocomposite with **f-MWCNTs@Pd/TiO$_2$**, we observed Tafel plots with similar slopes (indicative of a coherent electrocatalytic mechanism dominated by the Pd nanoparticles), but interestingly we measured 200 times higher TOF_0 value for the carbon-based nanohybrid (9460 H_2 h^{-1} vs. 47 H_2 h^{-1}, Fig. 3d). This behaviour was not totally unexpected and suggested a synergic contribution of both the inorganic **Pd/TiO$_2$** system and the nanostructured MWCNTs to the electrocatalytic performances. The enhancement of the TOF_0 and the lower overpotential needed to reduce water for the **f-MWCNTs@Pd/TiO$_2$** nanocomposite, were likely due to favourable changes in either the bulk and/or surface electronic properties of the TiO_2 crystals. Such a hypothesis was thoroughly investigated by Electrochemical Impedance Spectroscopy (EIS), a powerful tool that enables to specifically analyse the electrochemical interface between the nanohybrid electrocatalysts and the electrolytic solution.

The interfacial capacitance was measured at 1 kHz scanning the potential from 0.6 to -0.4 V (vs. RHE) and the data collected were then subjected to the classical Mott-Schottky analysis, eq. (1)

$$\frac{1}{C_{SC}^2} = \frac{2}{\varepsilon\varepsilon_0 N_D}\left(E - E_{fb} - \frac{k_B T}{e} \right) \qquad (1)$$

where C_{SC} is the capacitance of the nanostructured films (dominated, at the inspected frequency, by the space charge properties of TiO_2),[7] E is the applied potential, N_D the electron

carrier density, E_{fb} is the flat-band potential of TiO_2 and all the other symbols have their classical meaning. In Fig. 3a are shown the Mott-Schottky (M-S) plots for the various electrocatalysts. Extrapolation of the linear E-dependent part of the plots allowed us to estimate E_{fb}. E_{fb} values vary in the range from -0.22 V to -0.27 (vs. RHE), in good agreement with values reported in literature for TiO_2 in aqueous solutions.[8-10] Integration of **Pd/TiO₂** into the MWCNTs nanostructures did not therefore affect significantly the energy position of the semiconductor bands. By contrast, the N_D values differed significantly in the various cases, increasing by ~1600 times, i.e. from 1.1 x 10^{15} to 1600 x 10^{15} cm^{-3} on passing from **Pd/TiO_2** to **f-MWCNTs@Pd/TiO_2** (Supplementary Table 1). An analogous effect of a higher donor density caused by the presence of MWCNTs was recently observed also by Zhang and co-workers on ZnO nanowires.[11]
Interestingly, the N_D value was also strongly affected by changes in the amount of MWCNTs in the nanohybrids. This effect is shown in Supplementary Fig. 7 with a comparison between the M-S plots for **Ox-MWCNTs(20%)@Pd/TiO_2** and **Ox-MWCNTs(15%)@Pd/TiO_2**, whose slopes differ by a factor of two, corresponding, respectively, to N_D values of 6.4 x 10^{17} cm^{-3} and 3.3 x 10^{17} cm^{-3}. The capacity of the surface states (C_{ss}), proportional to the density of surface states, was obtained in the various samples from the plateau values in the M-S plots (Supplementary Table 1).
Notice that, in line with the decreased N_D, the nanohybrid with a lower MWCNTs content also displayed a decreased electrocatalytic efficiency, since the TOF_0 of **Ox-MWCNTs(15%)@Pd/TiO_2** (59 H_2 h^{-1}) was about 75% lower than that of **Ox-MWCNTs(20%)@Pd/TiO_2** (Supplementary Fig. 18 and Supplementary Table 1).

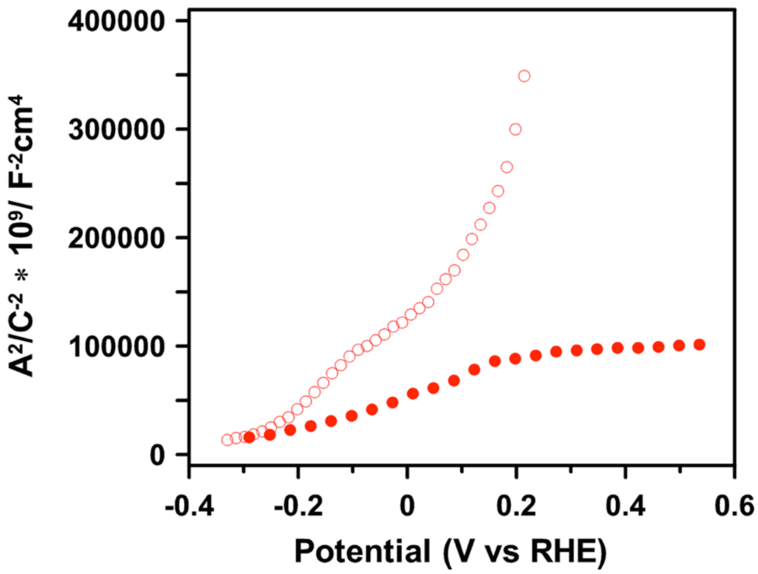

Supplementary Figure 8. Effect of calcination on the M-S plots. Mott-Schottky plots for **Tour-MWCNTs@Pd/TiO_2** (○) and **f-MWCNTs@Pd/TiO_2** (●) in phosphate buffer, pH = 7.4 at a fixed frequency of 1 kHz.

The effect of a thermal annealing on **Tour-MWCNTs@Pd/TiO_2** is reported in Supplementary Fig. 8, in which is highlighted how M-S plots change after the thermic treatment. From the figure it is evident the increase of N_D in **f-MWCNTs@Pd/TiO_2** (lower slope of the linear E-dependent region) and also the appearance of a region of extended surface states (at potentials more positive than 0.2 V vs. RHE), that was not observed before calcination.

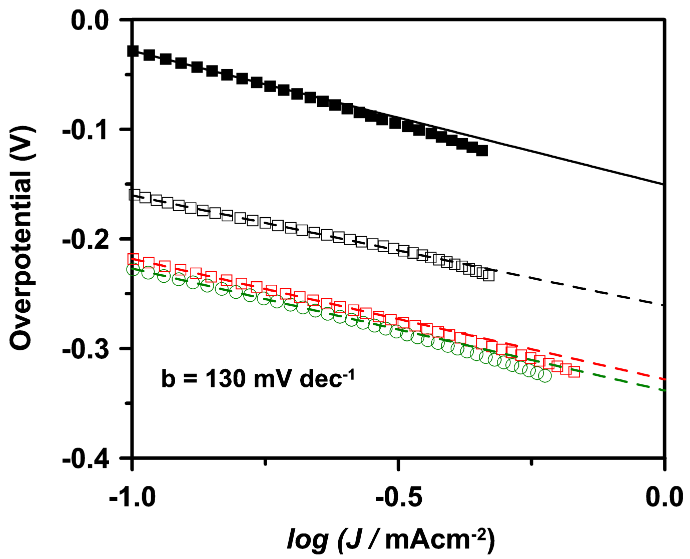

Supplementary Figure 9. Investigation of HER mechanism. Tafel plots for **f-MWCNTs@Pd/TiO₂** (■), **Ox-MWCNTs@Pd/TiO₂** (□), **Tour-MWCNTs@Pd/TiO₂** (□) and **Pd/TiO₂** (○)

The mechanism of the HER classically comprises three possible step-reactions [12]:

$$H_2O \leftrightarrows H^+ + OH^- + e^- \rightarrow H_{ad} + OH^- \qquad \text{Volmer} \qquad (2)$$

$$H^+ + H_{ad} + e^- \rightarrow H_2 \qquad \text{Heyrovsky} \qquad (3a)$$

$$H_{ad} + H_{ad} \rightarrow H_2 \qquad \text{Tafel} \qquad (3b)$$

Coupling of reaction (2) with either (3a) or (3b) results in the so-called Volmer-Heyrovsky or Volmer-Tafel routes. Elucidation of the prevailing mechanism is usually based on the inspection of the slopes of the η/log(i) plot (known as "Tafel plots") and of the log(i)/pH plot. In Supplementary Fig. 9 are reported the Tafel plots for the nanohybrid materials **f-MWCNTs@Pd/TiO₂** (black squares and line), **Tour-MWCNTs@Pd/TiO₂** (red empty sqaures and dashed line), **Ox-MWCNTs@Pd/TiO₂** (black empty squares and dashed line) and **Pd/TiO₂** (green empty dots and line). All the materials show similar Tafel slopes (~130 mV) that, together with the unitary slopes observed in the log(i)/log[H^+] (see Supplementary Fig. 10), would suggest that H_{ad} formation is the limiting step for HER in both cases.

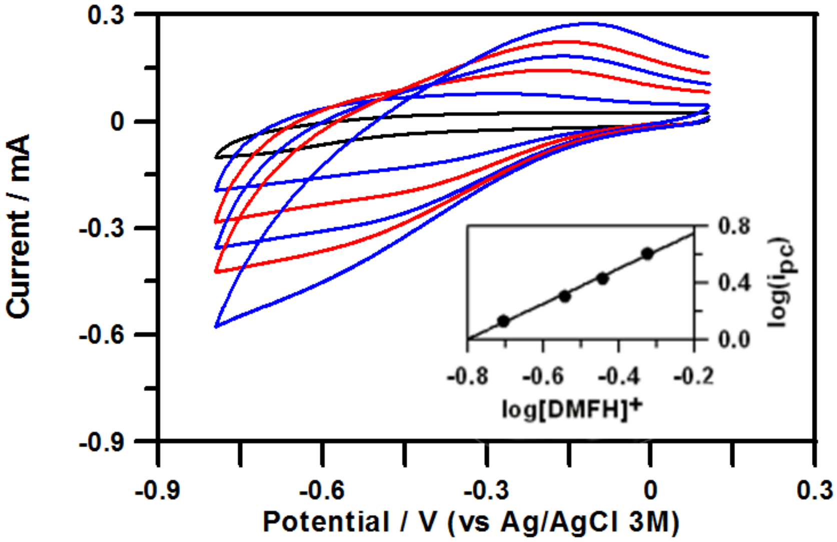

Supplementary Figure 10. Electrochemistry of f-MWCNTs@Pd/TiO_2 in non-aqueous media. CVs for **f-MWCNTs@Pd/TiO_2** drop-casted on ITO electrode in pure CH_3CN (black line) and after increasing additions of a specific proton source, 1:1 mol/mol [DMFH]OTf/DMF (resulting $[DMFH]^+$: 0.68, 1.36, 2.04, 2.72, 4.08 mM), in CH_3CN/ 0.08 M $TBAPF_6$ argon saturated solution. **Inset:** linear relationship between the peak cathodic current and $[DMFH^+]$ concentration. Scan rate = 0.05 Vs^{-1}. The diameter of the electroactive surface is 8 mm.

Preliminary investigations of the electrocatalytic properties of the **f-MWCNTs@Pd/TiO_2** nanohybrids deposited on ITO electrodes were carried out in an aprotic solvent (CH_3CN). In these conditions, the triflate salt of protonated dimethylformamide 1:1 mol/mol [DMFH]OTf/DMF (with $E^o_{H+/H2}$ = -0.285 V vs. Ag/AgCl)[13,14] was used as a proton source, and the HER kinetics were investigated by cyclic voltammetry (Supplementary Fig. 10). The reversible cathodic peak with onset at ~ -0.2 V and $E_{1/2}$ = -0.38 V (vs. Ag/AgCl), whose intensity increased linearly with proton concentration (inset of Supplementary Fig. 10), was attributed to proton reduction.

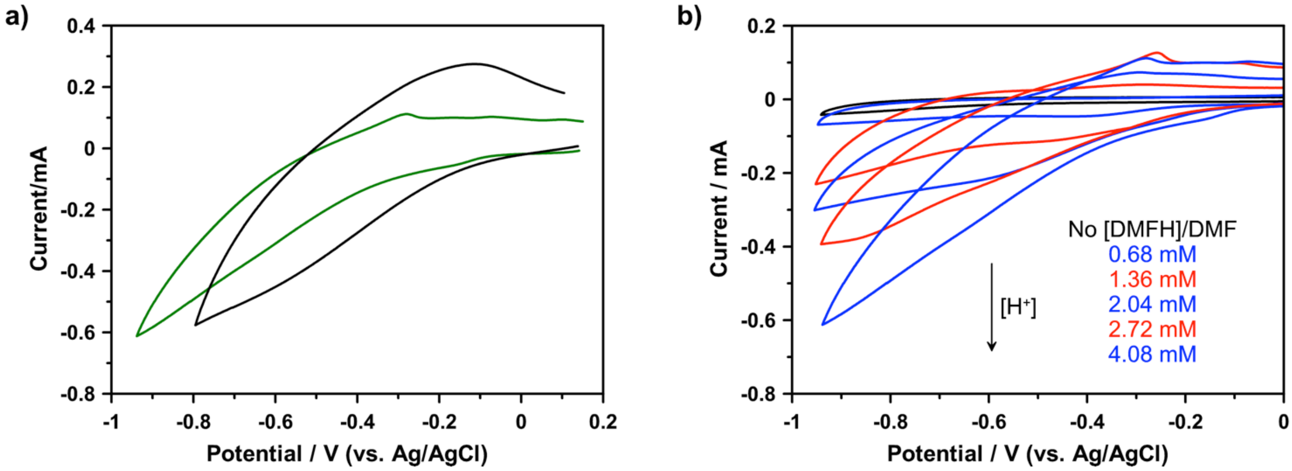

Supplementary Figure 11. Electrochemistry of the nanohybrids in non-aqueous media. (**a**) CV comparison of **f-MWCNTs@Pd/**$\mathbf{TiO_2}$ (black) and **Pd/**$\mathbf{TiO_2}$ (green) with a $[DMFH]^+$ concentration of 4.08 mM. (**b**) **Pd/**$\mathbf{TiO_2}$ in the pristine base solution (black line) and at various $[DMFH]^+$ concentrations (blue and red lines), as indicated: 0.68, 1.36, 2.04, 2.72 and 4.08 mM. The diameter of the electro-active surfaces is 8 mm. Scan rate 0.05 Vs^{-1} in CH_3CN /$TBAPF_6$ of an argon saturated solution.

Analogous behaviour was also observed in the case of the **Pd/**$\mathbf{TiO_2}$ electrodes (Supplementary Fig. 11b), although in this case the proton reduction peak is significantly shifted by almost 200 mV towards more negative potentials than in the case of **f-MWCNTs@Pd/**$\mathbf{TiO_2}$ (see Supplementary Fig. 11a for a direct comparison).

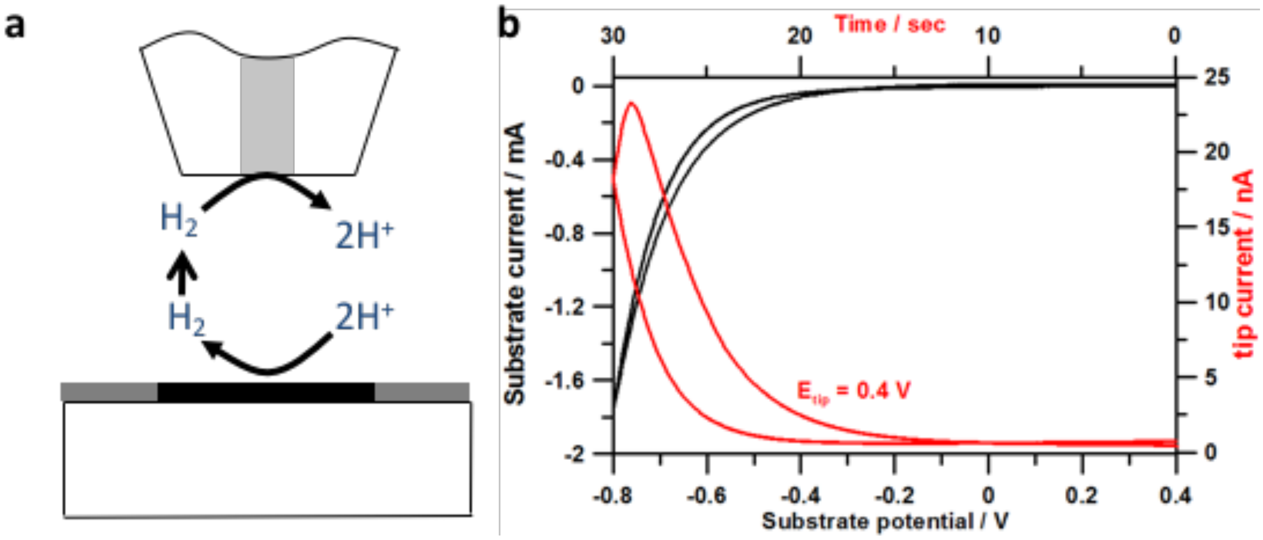

Supplementary Figure 12. Detection of the evolved H_2 by the SECM method (**a**) schematic representation of the SG/TC operative mode used to detect the hydrogen produced by the investigated nanohybrids. As substrate electrodes we used the different nanohybrids, while the tip was a Pt microelectrode. (**b**) Typical SG/TC experiment in phosphate buffer solution (pH 7.4). Tip: Pt microelectrode (red line) (15 μm), substrate**: f-MWCNTs@Pd/TiO_2** deposited on a SPEs (black line)**.** Experimental conditions: Ar saturated solution. The tip was held at a constant potential of 0.4 V (vs. RHE), while the substrate was scanned at a rate of 0.1 Vs^{-1} from 0.6 V to -0.8 V vs. RHE.

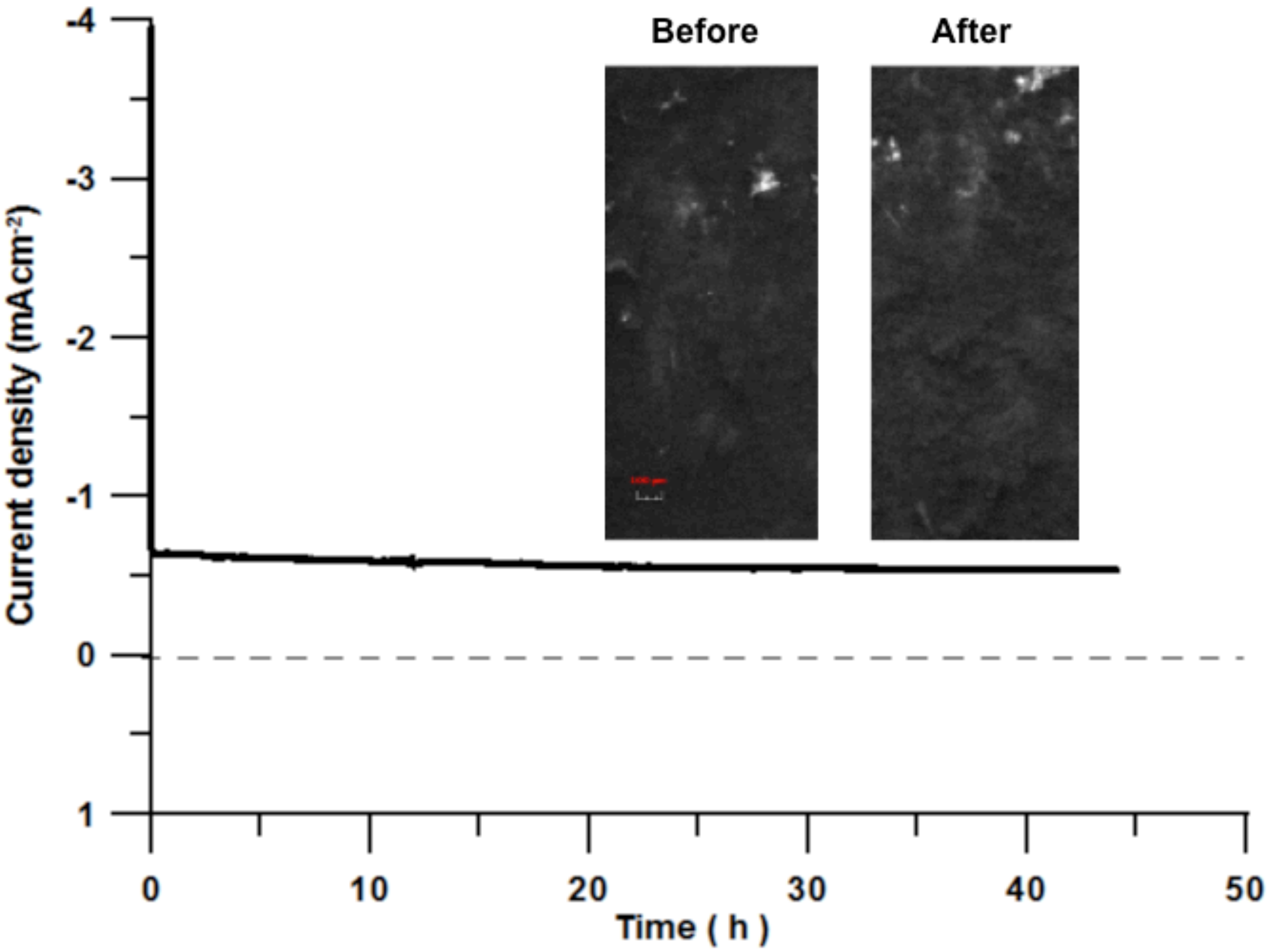

Supplementary Figure 13. Stability test during hydrogen evolution. Chronoamperometry in phosphate buffer 0.1 M (pH 7.4) over 40 h after the deposition of the nanocomposites **f-MWCNTs@Pd/TiO$_2$**. Overpotential -0.15 V. The inset shows optical images of the electrode surface before and after electrolysis.

The high stability of the **f-MWCNTs@Pd/TiO$_2$** nanohybrid was tested over 45 h of extended electrolysis observing a slight decrease, < 15%, of the cathodic current under HER condition (Supplementary Fig. 13).

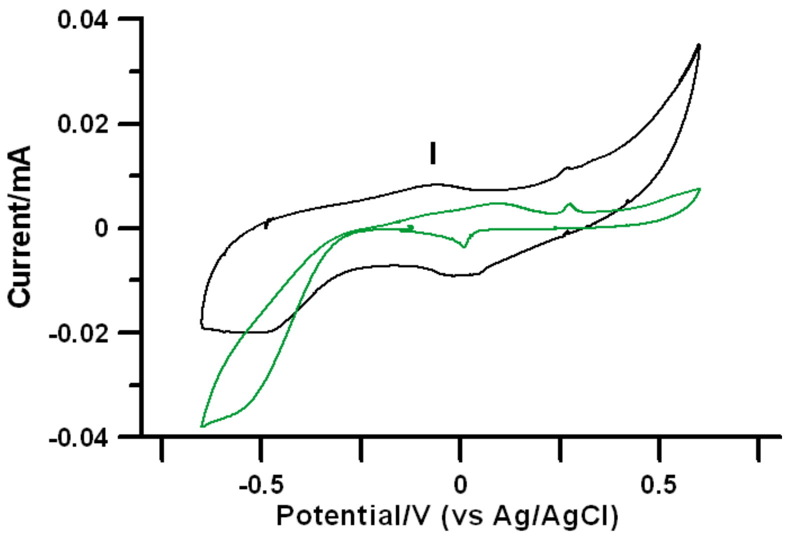

Supplementary Figure 14. Determination of electroactive Pd. CVs for **f-MWCNTs@Pd/TiO$_2$** (black) and **Pd/TiO$_2$** (green) drop-casted on SPEs,in NaOH 2 M. The electrode surface has a diameter of 4 mm.

The amount of electroactive Pd (Q_{Pd}) in the nanocomposites was estimated by CV, following a reported procedure.[2,3] The approach involves the measurement of the charge related to the formation of a surface Pd-oxide layer onto the Pd nanoparticles during a voltammetric scan towards positive potentials, in a 2 M NaOH aqueous solution.

$$Pd + 2\,OH^- \rightarrow PdO + H_2O + \,2\,e^- \qquad (9)$$

Integration of the charge underneath the PdO formation peak (labelled with I in Supplementary Fig. 14) revealed the amount of Pd electrochemically active.

The Q_{Pd} values thus obtained for the various nanohybrids are summarized in Supplementary Table 1 and used used for TON/TOF calculation (see Supplementary Note 3).

Considering the mean size distribution of the spherical Pd NPs and the charge surface densities for Pd oxidation,[2] we estimated that roughly 8-20% of the total Pd atoms on the surface of the NPs are available for electrochemical reactions depending on the measured sample.

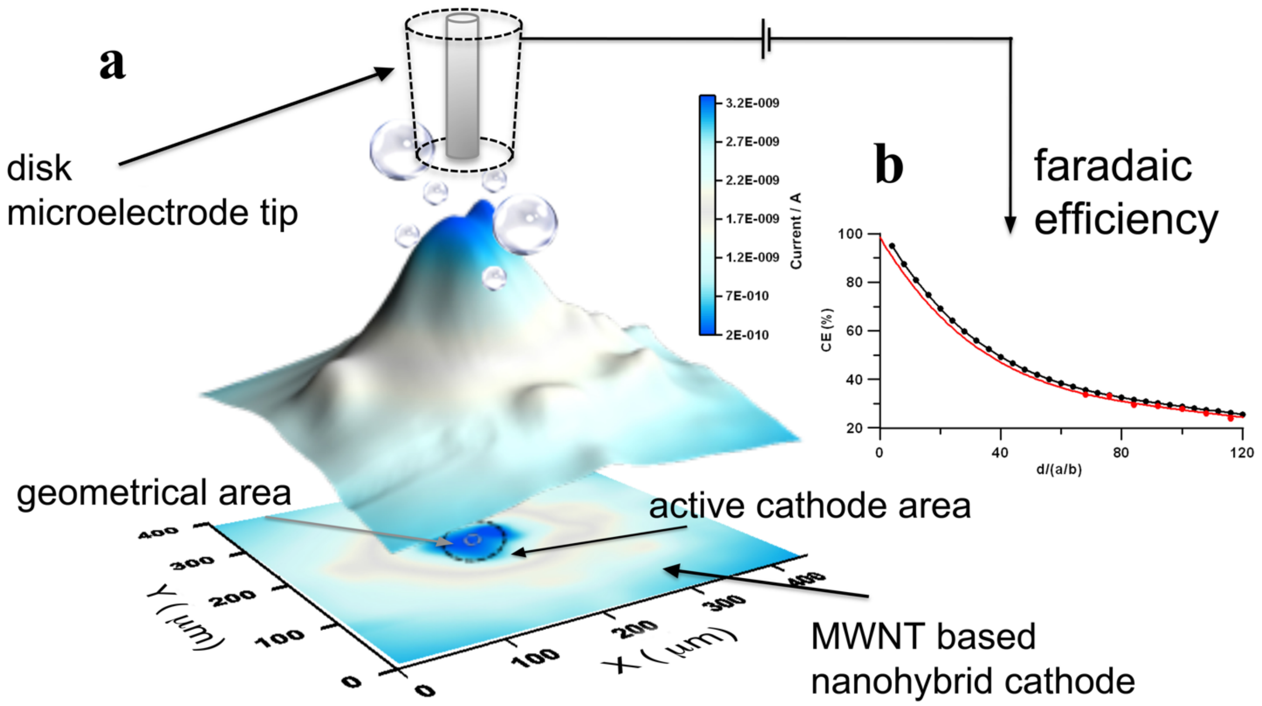

Supplementary Figure 15. SECM method for FE quantification. (**a**) schematic representation of the SG/TC experiment used to evaluate the faradaic efficiency of the cathodic process. Relevant geometric parameters: d = 25 μm is the distance between the tip and the substrate, a = 15 μm is the radius of the Pt SECM tip (embedded in a glass tube with an outer diameter of 110 μm, not in scale), b = 50 μm is the radius of the electroactive area of a representative MWCNTs-based nanohybrid film. The underlying UME, used to contact the nanocomposite film, is indicated in light gray. (**b**) experimental CE values (red dots) compared to the corresponding CE_{max} values obtained from eq. 8 (black dots and line). Experimental conditions: Ar-saturated phosphate buffer (pH = 7.4), E_{tip} = 0.4 V vs. RHE, E_{sub} = -0.6 V, vs RHE.

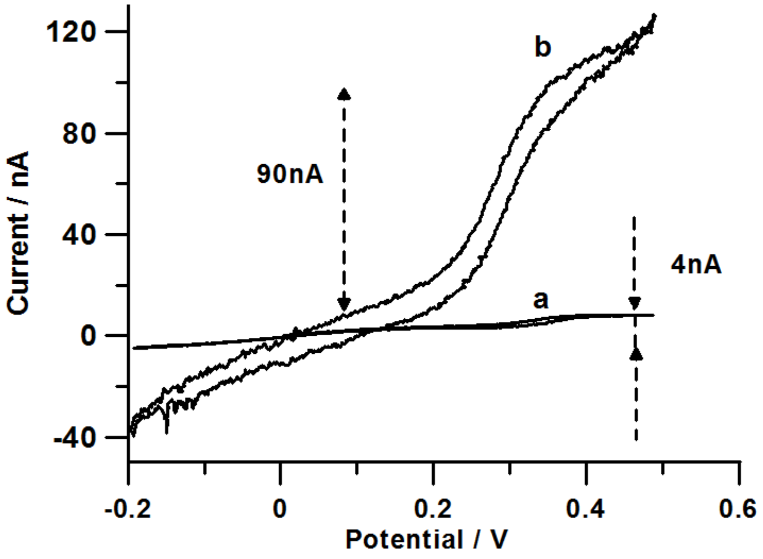

Supplementary Figure 16. Determination of electroactive area by CV. Cyclic voltammetric curves of 1 mM Ferrocenemethanol for the substrate electrode (diameter 25 μm) before (**a**) and after (**b**) the modification with **f-MWCNTs@Pd/TiO$_2$** in 100 mM $LiClO_4$ aqueous solution. Scan rate 0.05 Vs^{-1}. Potentials are reported vs. Ag/AgCl/KCl 3M.

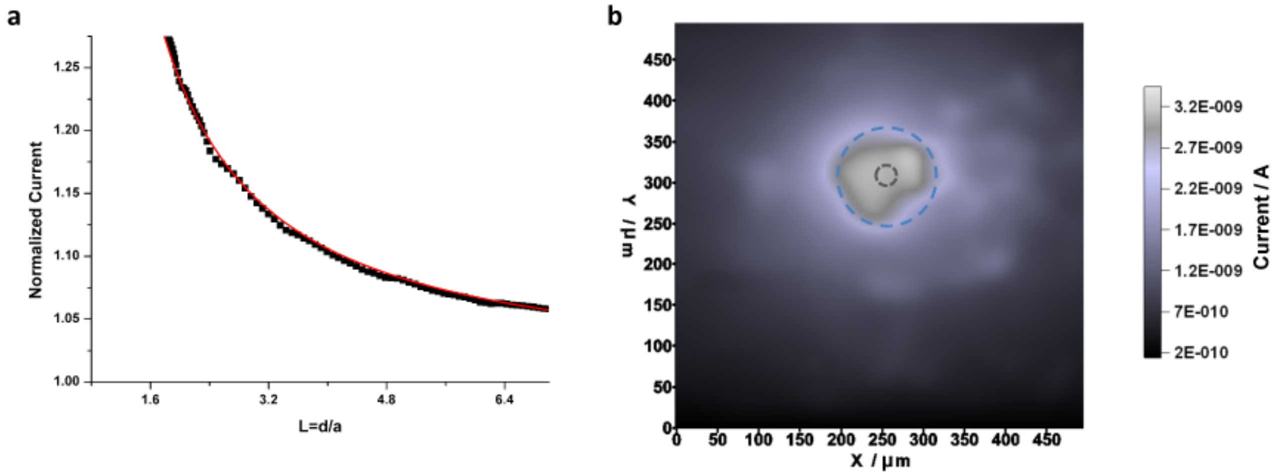

Supplementary Figure 17. Investigation of electroactive area by SECM. (**a**) Approach curve in a feedback mode for the platinum tip microelectrode (a = 15 μm) in 1 mM Ferrocenemethanol/ 100 mM $LiClO_4$ aqueous solution. Tip potential 0.4 V. (**b**) SG-TC in the same experimental conditions that displays the real substrate geometry after the deposition of **f-MWCNT@Pd/TiO_2**. E_{tip} = 0 V, E_{sub} = 0.45 V. Potentials are reported vs. Ag/AgCl/KCl 3M.

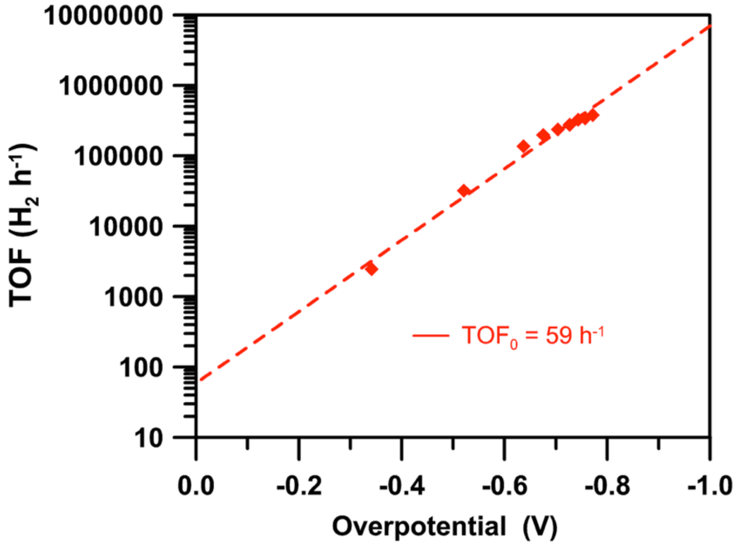

Supplementary Figure 18. TOF for Ox-MWCNTs(15%)@Pd/TiO_2. TOF as a function of η. η = E_{app} – E^0 – i_{ss} × R_S where E_{app} is the applied potential, E^0 is the thermodynamic potential corrected for the pH (E^0 [H^+/H_2] = -0.21 – 0.059 × pH = -0.65 V vs Ag/AgCl and i_{ss} × R_S is the correction for the ohmic drop (i_{ss} is the stationary state current measured during the potential step and R_S the uncompensated resistance). TOF_0 is the extrapolated turnover frequency at zero overpotential (TOF at η = 0 V).

The electrocatalytic performances of **Ox-MWCNTs(15%)@Pd/TiO_2** are reported in Supplementary Fig. 18. Extrapolation of the TOF-η semilogaritmic plot at η = 0 V, gives the turnover frequency at zero overpotential (TOF_0). This value can be used as an optimal descriptor of the intrinsic catalytic activity for different electrocatalytic materials. For **Ox-MWCNTs(15%)@Pd/TiO_2** the TOF_0 was estimated to be 59 H_2 h^{-1}, whereas the analogous nanohybrid **Ox-MWCNTs(20%)@Pd/TiO_2**, having a higher concentration of MWCNTs, displayed a much higher TOF_0 of 251 H_2 h^{-1}. The fundamental role of MWCNTs was again highlighted by the correlation between electrochemical activity and the content of MWCNTs.

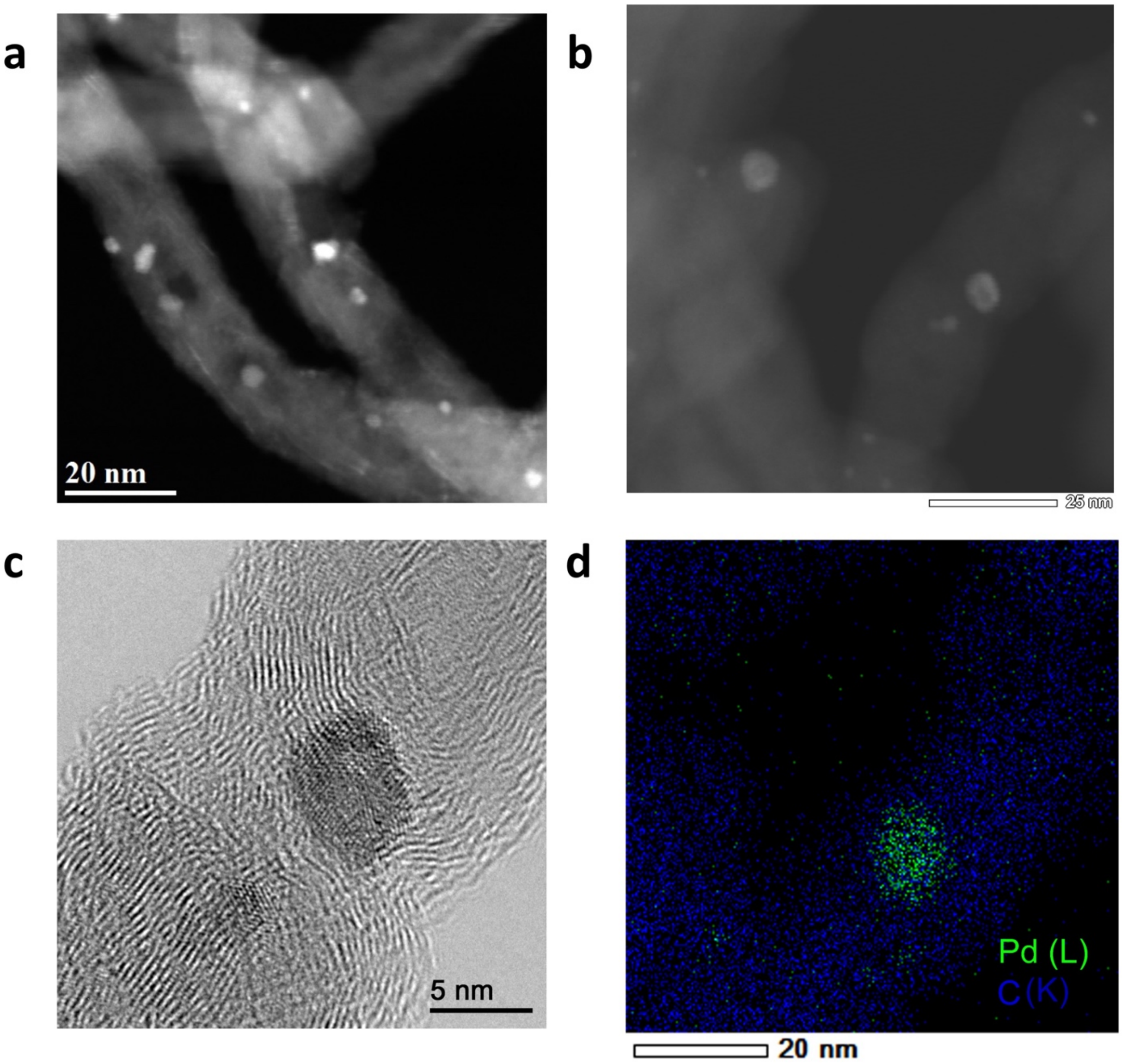

Supplementary Figure 19. **Characterization of f-MWCNTs@Pd.** (**a**) Representative STEM-HAADF image of the **f-MWCNTs@Pd.** (**b**) STEM-HAADF image of some Pd NPs with the corresponding (**c**) HRTEM image and (**d**) EDXS map.

We compared **f-MWCNTs@Pd/TiO_2** with **f-MWCNTs@Pd**, a model system having the Pd NPs directly in contact with the MWCNTs and not embedded into the TiO_2 shell**. f-MWCNTs@Pd** was synthesized as described in the Supplementary Note 1. Supplementary Fig. 19 shows representative TEM images of such system, where Pd nanoparticles are clearly observed on the **f-MWCNTs**.

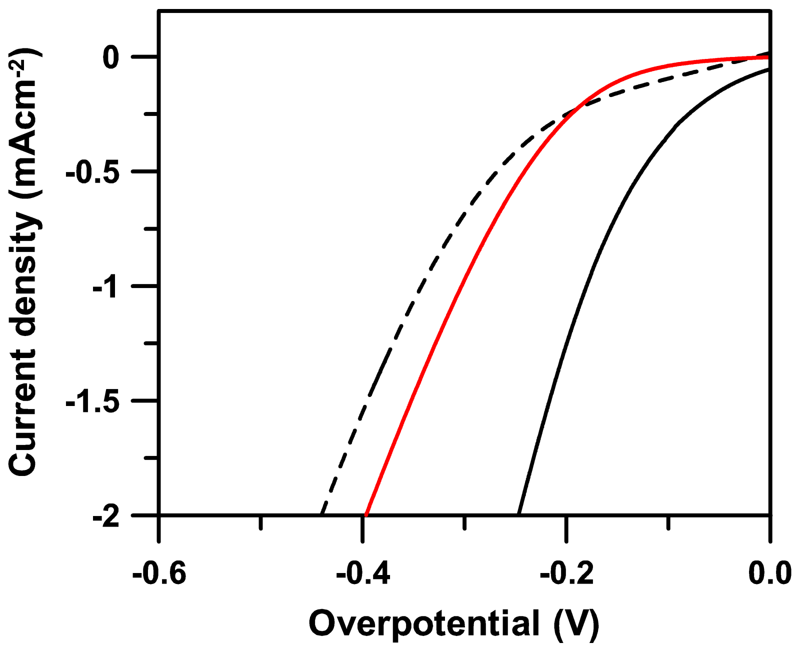

Supplementary Figure 20. HER activity of physical mixtures. LSV in phosphate buffer (pH 7.4) after the deposition of the same amount of **f-MWCNTs@Pd/TiO_2** (black solid line), and the mixtures of **f-MWCNTs@TiO_2 and Pd/TiO_2** (red solid line), and **Ox-MWCNTs and Pd/TiO_2** (black dashed line). Scan rate = 2 mVs^{-1}.

The higher catalytic activity towards HER due to synergic effects between the various building blocks is also revealed by a comparison of **f-MWCNTs@Pd/TiO_2** with physical mixtures of the various components (Supplementary Fig. 20). Mixtures of **Ox-MWCNTs** and **Pd/TiO_2**, and also **Ox-MWCNTs/TiO_2** and **Pd/TiO_2** do not reach the same activity of the nanohybrid **f-MWCNTs@Pd/TiO_2**. In Supplementary Fig. 20 are reported the LSVs in phosphate buffer for the various mixtures, that have been deposited on the substrate electrodes in thin films with the same nominal coverage.

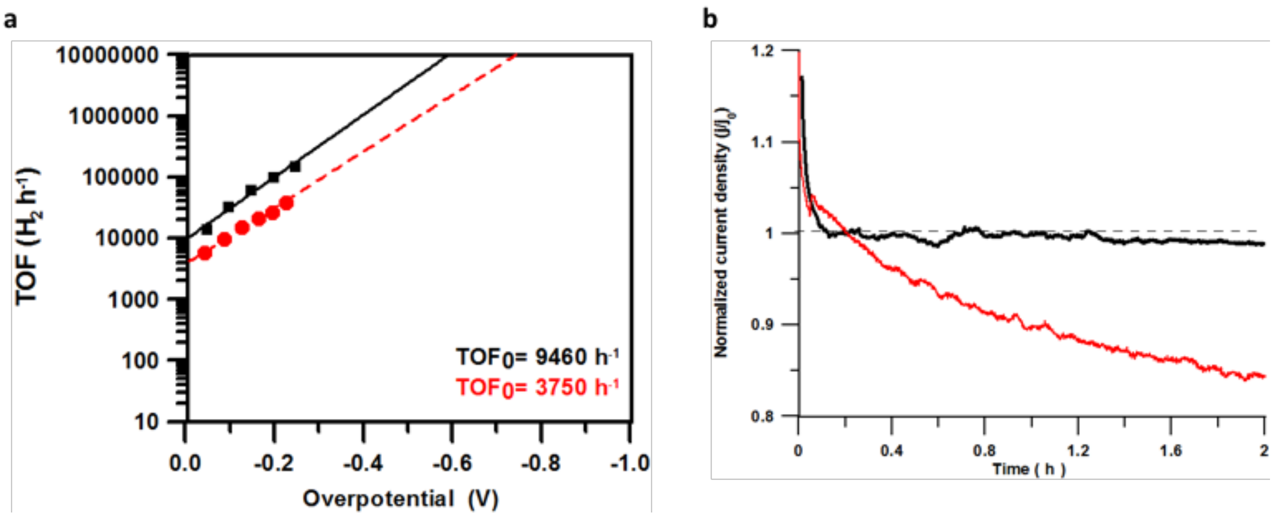

Supplementary Figure 21. Comparison between the electrocatalytic performances of f-MWCNTs@Pd and f-MWCNTs@Pd/TiO₂. (**a**) TOF as a function of η for **f-MWCNTs@Pd** (red line) and **f-MWCNTs@Pd/TiO₂** (black line). $\eta = E_{app} - E^0 - i_{ss} \times R_S$ where E_{app} is the applied potential, E^0 is the thermodynamic potential corrected for the pH (E^0 [H^+/H_2] = -0.21 – 0.059 × pH = -0.65 V vs Ag/AgCl and $i_{ss} \times R_S$ is the correction for the ohmic drop (i_{ss} is the stationary state current measured during the potential step and R_S the uncompensated resistance). TOF_0 is the extrapolated turnover frequency at zero overpotential (TOF at η = 0 V). (**b**) Chronoamperometry in phosphate buffer 0.1 M (pH 7.4) over 2 h after deposition of **f-MWCNTs@Pd** (red line) and **f-MWCNTs@Pd/TiO₂** (black line). Applied overpotential -0.15 V. The currents were normalized for J_0 (the extrapolated current density at t = 0 sec) in order to directly compare the stability of the two materials.

The TOF were calculated as fully explained in Supplementary Note 3. Extrapolation of the TOF at zero overpotential (TOF_0) gives for **f-MWCNTs@Pd** values that are more than two times lower than **f-MWCNTs@Pd/TiO₂** (9460 h^{-1} vs. 3750 h^{-1}, Supplementary Fig. 21a). We also compared the chemical and mechanical stability of these two nanocomposites by chronoamperometry, as evidenced in Supplementary Fig. 21b. While **f-MWCNTs@Pd/TiO₂** shows a stable HER current (15% decay in 45 h, see also Supplementary Fig. 13) in the case of **f-MWCNTs@Pd** the current decays by ~15% of the initial value within the first 2 hours of potentiostatic electrolysis**.** This behaviour highlights the essential role of the TiO_2 layer to increase the stability of the catalyst.

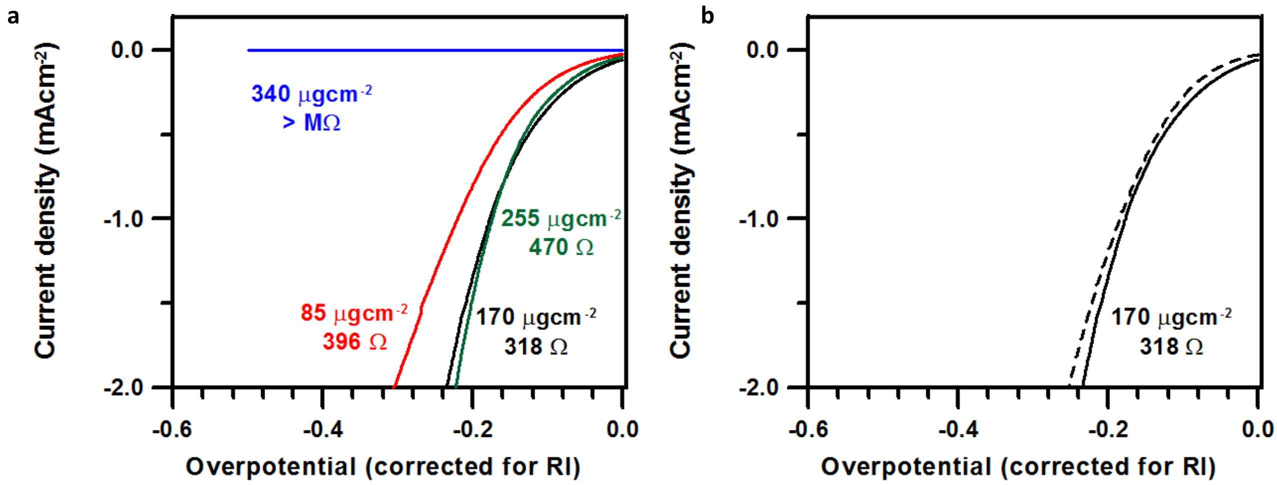

Supplementary Figure 22. Catalyst loading optimization. (**a**) Linear sweep voltammetry curves in phosphate buffer (pH 7.4) after the deposition of different amounts of **f-MWCNTs@Pd/TiO$_2$:** 85 µgcm^{-2} (red line), 170 µgcm^{-2} (black solid line), 255 µgcm^{-2} (green line), 340 µgcm^{-2} (blue line). (**b**) Two independent LSV experiments with a loading of 170 µgcm^{-2} (full and dashed lines). Scan rate = 2 mVs^{-1}. The resistance of the deposits measured by electrochemical impedance spectroscopy is also reported.

We optimized the catalysts loading by combining the optimal amount of the active material with the conductivity of the sample. We found the best performances using a loading of 170 µg cm^{-2}. This amount of catalytic material results low when compared with some literature data,[6] and the estimated total amount of noble metal is in order of 1 µg of Pd. In Supplementary Fig. 22a are reported the HER performances as function of different loadings, and the associated uncompensated resistance for the various deposits. We choose here the best compromise that minimizes the resistance, while maximising the HER activity.

Supplementary Table 1: Figures of merit of the various nanohybrids obtained from the electrochemical characterization.

Catalyst [a]	η @ -1 $mA\ cm^{-2}$ (V) [b]	Slope ($mV\ dec^{-1}$)	Q_{Pd} (μC)	TOF_0 ($H_2\ h^{-1}$) [c]	C_{ss}/A ($\mu F cm^{-2}$) [d]	$N_D \times 10^{15}$ (cm^{-3})
Pd/TiO_2	-0.37	130	6.4	47	200	1.1
Ox-MWCNTs(20%)@Pd/TiO_2	-0.31	130	2.7	251	8200	640
Ox-MWCNTs(15%)@Pd/TiO_2	-0.35	130	9.3	59	1200	330
Tour-MWCNTs@Pd/TiO_2	-0.35	130	7.0	133	-	670
f-MWCNTs@Pd/TiO_2	-0.17	130	3.0	9460	1500	1600

a) Nanohybrids used for cathode fabrication. b) overpotential for HER in phosphate buffer solution (pH = 7.4), measured for a cathodic current of -1 $mAcm^{-2}$. c) turnover frequency determined by extrapolation at η = 0 V. d) surface states' capacitance obtained from the Mott-Schottky plots

Supplementary Table 2. Comparison between the electrochemical performances of **f-MWCNT@Pd/TiO_2** with other recently reported, highly active, HER electrocatalysts that operate at neutral pH.

Catalyst	J_0 (A cm^{-2})	TOF_0 (H_2 h^{-1})	Tafel slope (mV dec^{-1})	Reference
**f-MWCNTs@Pd/TiO_2**	**6.0 × 10^{-5}**	**84**	**130**	**This work**
**MWCNT/Co**	3.1 × 10^{-7}	80000 @ η = 600 mV	160	Nature Chem., 2013, 5, 48
**H_2-CoCat/FTO**	3.1 × 10^{-6}	80 @ η = 385 mV	140	Nature Mater., 2012, 11, 802
**CoN_x/C**	7 × 10^{-5}	30.2 #	-	Nature Comm., 2015, 6:7992
**Amorphous MoS_2**	-	9.7 §	-	Chem. Sci., 2011, 2, 1262
**$CuMoS_4$**	4 × 10^{-5}	1 *	95	EES, 2012, 5, 8912
**Co NPs-NRCNTs**	1.0 × 10^{-5}	55 @ η = 330mV	-	Angew. Chem. Int. Ed., 2014, 53, 4372
**CoP**	2.9 × 10^{-4}	-	93	J. Am. Chem. Soc., 2014, 136, 7587
**FeS NPs**	6.6 × 10^{-4}	-	150	ACS Catalysis, 2014, 4, 681
**Ni/MoS_3**	1.0 × 10^{-5}	-	86	Chem. Sci., 2012, 3, 2515

The TOF herein reported were calculated considering the entire catalyst loading on the various electrodes.

§ Derived from fitting of data reported in Ref. 14 and by extrapolation at η = 0 V.
# Derived from fitting of data reported in Ref. 15 and by extrapolation at η = 0 V.
* Derived from fitting of data reported in Ref. 16 and by extrapolation at η = 0 V.

Supplementary Note 1: Synthesis of the various nanohybrids

Synthesis of Ox-MWCNTs

150 mg of pristine MWCNTs (NanoAmor, 20–30 nm diameter, 0.5–2 μm length) were dispersed in a concn H_2SO_4/concn HNO_3 mixture 3:1 v/v (100 mL) by sonication (6 h at 30−50 °C) and magnetic stirring (12 h at 50 °C). The suspension was washed six times by filtration; twice with water (250 mL), twice with NaOH (0.1 M, 250 mL), once with DMF (250 mL), and once with THF (250 mL).

Synthesis of Tour-MWCNTs

100 mg of pristine MWCNT (NanoAmor, 20–30 nm diameter, 0.5–2 μm length) were dispersed in 100 mL of H_2O by sonication for 20 minutes. Then the p-amino-benzoic acid (3.3 equivalents respect to carbon moles in the MWCNTs) was added and mixed by sonication for further 10 minutes. The mixture was put under vigorous stirring and 2.25 mL of isopentyl nitrite were added just before start the heating treatment at 80 °C for 6 h under reflux. Caution: diazonium salts should be handled with care since they can be explosive. The mixture was naturally cooled down at room temperature and collected by filtration on a 0.1 μm polytetrafluoroethylene Millipore membrane. Finally, the material was washed with DMF, MeOH, H_2O, EtOH and Et_2O sonicating the solid for 5 minutes in each solvent and dried overnight at 80 °C.

Synthesis of f-MWCNTs@Pd

An appropriate amount of Tour-MWCNTs is dispersed in EtOH via 30 minutes of sonication in order to have a 0.5 mg mL^{-1} dispersion. A solution of NaOH (1M) is added until the pH reaches ~12. A solution of an appropriate amount of K_2PdCl_4 (in order to have a final loading of 1.5 wt % of Pd) is added while sonicating the MWCNT dispersion. The mixture is then stirred at 100 °C for 2 hours, the solid recovered by filtration on a 0.45 μm polytetrafluoroethylene Millipore membrane and washed twice with EtOH/H_2O and once only with EtOH. The solid is dried at 85 °C overnight. Part of the solid is then subjected to calcination at 350 °C for 5 hours (+3 °C min^{-1}; -4.5 °C min^{-1}) to obtain the final **f-MWCNTs@Pd** catalyst.

Synthesis of f-MWCNTs@Pd/TiO_2 (with Ox- or Tour-MWCNTs)

The synthesis is described for the 20% MWCNTs composition. The f-MWCNTs (20 mg of **Ox-MWCNTs** or **Tour-MWCNTs**) were dispersed in absolute ethanol (EtOH mL/*f*-MWCNT mg ratio: 2.5) by sonication for 30 minutes. Meanwhile a Pd-MUA THF solution (containing 1.5 mg of Pd) was slowly added to a THF solution of Ti(O-*n*-Bu)$_4$ (containing 78.5 mg of TiO_2 respectively). Then the **Pd@TiO_2** precursors solution was slowly added under sonication to the *f*-MWCNTs dispersion and the mixture was further sonicated for 30 minutes. Finally, a 10% solution of H_2O in EtOH (Ti(*n*-OBu)$_4$ /H_2O molar ratio: 1/120) was dropped and the mixture sonicated for 30 minutes. The materials were collected by filtration on a 0.45 μm polytetrafluoroethylene Millipore membrane, washed with ethanol and dried overnight at 80 °C. In order to eliminate the organic ligands and improve the crystallization of the TiO_2 the catalysts were subjected to calcination at 350 °C for 5 hours (+3 °C min^{-1}; -4.5 °C min^{-1}). As shown by typical thermogravimetric analysis (TGA) profiles from a fresh (**Ox-MWCNTs@Pd/TiO_2**) and calcined (**f-MWCNTs@Pd/TiO_2**) samples (Supplementary Fig. 6), respectively, the weight loss at around 230 °C, characteristic of the organic moieties and present in the former, disappears completely in the latter. Such behavior confirms the successful removal of the organic groups (the functional groups of MWCNTs and the organics deriving from the metal precursors).

Supplementary Note 2: Experiments in aqueous media

The nanocomposites were deposited by drop-cast onto amorphous carbon Screen Printed Electrodes (SPEs) and all the experiments were carried out in Ar-saturated solutions. The cathodic currents observed at potentials ≤ -0.1 V (vs RHE) were unequivocally attributed to the reduction of water by detecting the evolved hydrogen using a Scanning Electrochemical Microscope in a Substrate-Generation Tip-Collection mode (SG-TC, Supplementary Fig. 12 and Supplementary Note 4). This instrumentation allowed us to place a Pt microelectrode tip at a very short distance from the nanostructured catalysts, using the micrometric tip as a hydrogen probe. As far as we biased the Pt tip at 0.4 V vs RHE the only faradaic process that we might observe was the oxidation of H_2 (generated at the substrate) to H^+ (Supplementary Fig. 12a).

From the results we could then confirm H_2 as the product of the electrocatalytic HER that occurs at the bottom electrode. In Supplementary Fig. 12b is displayed the simultaneous hydrogen evolution (black line) and hydrogen detection (red line) during a voltammetric scan of a representative **f-MWCNTs@Pd/TiO$_2$** electrode (substrate). An extensive explanation of the procedure of substrate-generation tip-collection (SG/TC) followed to determine the actual faradaic efficiency is given in the Supplementary Note 4.

Supplementary Note 3: Quantification of the catalytic activity

A deeper insight in the electrocatalytic behaviour of the nanohybrid was obtained through the quantitative analysis of the evolved H_2 as a function of the applied potential, carried out performing a sequence of chronoamperometric experiments in which the electrode potential was stepped back and forth between 0.6 V and different negative values (ranging from 0.6 to -0.8 V vs. RHE, Fig. 3c). Each potential step was held constant until steady currents were observed (usually 200 seconds) and measured. Experiments were carried out under similar conditions with either **Pd/TiO$_2$**, **f-MWCNTs@Pd/TiO$_2$** and the other nanohybrids herein investigated. Importantly, all catalysts displayed great chemical and mechanical stability (even under massive hydrogen evolution conditions). The catalytic currents at any potential are stable and reproducible, without any significant loss of activity after repetitive cycling and after more than 45 h operation (Supplementary Fig. 13).

Given the difficulty to find a unique and valid methodology to compare the activity of nanostructured electrocatalytic systems, in this work it has been developed a new approach for turnover number (TON) and frequency (TOF) calculation that is particularly suited to compare the different catalytic systems herein studied. The strategy adopted is entirely based on electrochemical techniques, and the derivation of the various factors that influence the TOF is simply obtained by classical cyclic voltammetry (CV) and chronoamperometry (CA) techniques, together with a SECM-based approach for the determination of the Faradaic Efficiency (FE).

The TON (= moles of product/moles of catalyst, Eq. 4) and TOF (= TON/time, Eq. 5) are calculated from Faraday law relating the "moles of product" to the charge exchanged during each potential step in CA experiments, while the normalization for the "moles of catalyst"

accounts for the amount of electroactive Pd present in the nanohybrids. The moles of products considered are those produced only by the catalytic Pd sites, and that explain the contribution of the metal-free model system **Ox-MWCNTs@TiO_2** on top of Eq. 4, where its charge is subtracted to that of **Ox-MWCNTs@Pd/TiO_2**. The same concept applies also for **Tour-MWCNTs@Pd/TiO_2** and **Tour-MWCNTs@TiO_2**. The so-obtained TON are then weighed for the FE of the cathodic process (i.e., the fraction of the overall cathodic current that may be actually attributed to the HER) as determined by SECM. The procedure is explained in detail in Supplementary Note 4. Such approach allows to normalize the activity on an electroactive area basis, like the ECSA method, obtaining an intrinsic descriptor of the electrocatalytic activity.[18]

$$\mathrm{TON}_\eta = \frac{\left(Q_{H_2}^{\eta,\,\mathrm{MWCNT@Pd/TiO_2}} - Q_{H_2}^{\eta,\,\mathrm{MWCNT@TiO_2}}\right)}{Q_{\mathrm{Pd}}} \times \mathrm{FE} \qquad (4)$$

$$\mathrm{TOF}_\eta = \frac{\mathrm{TON}_\eta}{\mathrm{time}} \qquad (5)$$

The amount of electroactive catalyst (Q_{Pd}) was determined accordingly to reported methods [2,3], and was expressed as the charge related to the complete oxidation of Pd nanoparticles' surface. For **Pd/TiO_2** and **f-MWCNTs@Pd/TiO_2** the corresponding Q_{Pd} charges have been estimated to be 6.4 μC and 3.0 μC, respectively (see Supplementary Table 1). Finally, the FE of HER is taken in consideration to weight the real contribution of HER and not to overrating the TON/TOF of the nanohybrids.

This approach allowed us to do a comprehensive evaluation of the catalytic activity, and has been adopted for all the systems herein studied. It is worth mentioning that in the case of **Pd/TiO_2** we did not subtract the contribution of the Pd-free TiO_2, so the reported TOF_0 for **Pd/TiO_2** is an upper measure of its catalytic activity. Despite this fact, however, the electrochemical performance of **Pd/TiO_2** fell behind those of all the C-based nanocomposites, for which we considered only the contribution of Pd nanoparticles to HER, thus giving a lower value of their TOF_0 estimation.

Such procedure was particularly suited for a comparative description of the different catalytic systems herein studied, and allowed to highlight the effects on the electrochemical activity of the thermal annealing and of the different chemical functionalization of f-MWCNTs.

The TON/TOF calculations were carried out for **f-MWCNTs@Pd/TiO_2,** the model system **Pd/TiO_2, Ox-MWCNTs@Pd/TiO_2, Tour-MWCNTs@Pd/TiO_2** and **Ox-MWCNTs(15%)@Pd/TiO_2** (Fig. 3d and Supplementary Fig. 18). The Tafel-like behaviour, with no deviation from linearity up to η = -0.9 ÷ -1.0 V observed in all cases, is a consequence of the

exponential dependence of the quasi-stationary currents with overpotential, and confirms the attainment of a well-behaved ohmic contact of the catalytic nanohybrids to the conducting SPE substrates. The linear TOF-η relationship finally allowed for a straightforward extrapolation of the TOF_0 intercept value.

The outstanding electrocatalytic performance of the **f-MWCNTs@Pd/TiO$_2$** nanohybrid was documented by a TOF_0 of 9460 H_2 h^{-1} that lists it among the most efficient heterogeneous C-based HER electrocatalysts at neutral pH so far reported (Supplementary Table 2 and Fig. 4 in the manuscript). For the sake of comparison, **Ox-MWCNTs@Pd/TiO$_2$** displayed a TOF_0 of 251 H_2 h^{-1}, **Tour-MWCNTs@Pd/TiO$_2$** of 133 H_2 h^{-1}, **Pd/TiO$_2$** of 47 H_2 h^{-1} and **Ox-MWCNTs(15%)@Pd/TiO$_2$** of 59 H_2 h^{-1}.

Given the difficulty to find TOF values reported in terms of electrochemically active catalysts, and in order to compare the performances of **f-MWCNTs@Pd/TiO$_2$** with those of state-of-the-art electrocatalysts that operate in similar conditions of neutral electrolytes, we also normalized Eq. 4 for the entire Pd loading on the electrodes, considering that all Pd contributed to electrocatalysis. These "mass-normalized" TOF values are reported in Fig. 4 in the manuscript.

Supplementary Note 4: Quantification of the Faradaic Efficiency by the SECM method

The evaluation of the TON and TOF for the various electrocatalytic nanostructured films investigated in this work can be directly evaluated from the cathodic currents measured at the conducting substrate, once the faradaic efficiency (FE) of the process is known.

In order to determine the FE of HER, we took advantage of an experimental procedure based on the use of the Scanning Electrochemical Microscope (SECM), in a so-called SG (substrate generator)/TC (tip collection) experiment (Supplementary Fig. 12a). This operating mode was recently described for the rapid and high throughput screening of various electrocatalysts,[19] e.g. for the oxygen reduction reaction.[20] For our purpose, the main advantage of this technique is the ability to position with micrometric precision a ultramicroelectrode (UME) probe tip very close to the electrode surface (substrate).[21] In this case the probe was a Pt UME anode, biased at 0.4 V (vs RHE) so as to measure the hydrogen evolved from the surface, biased at different

negative potentials (herein reported the case for -0.6 V vs RHE), i.e. in a typical generator-collector configuration.[22] From the ratio between the current density measured at the tip (j_{tip}), and at the substrate (j_{sub}), the F.E. was then easily obtained (eq. 6) using specific working curves,[20] that define the maximum collection efficiency (CE_{max}) for different experimental set-up:

$$FE = \frac{J_{tip}/J_{sub}}{CE_{max}} \qquad (6)$$

Some important geometric parameters cooperate to define the working curve CE_{max} (eq. 8), such as the tip radius (a), the substrate radius (b) and the tip-substrate distance (d). Another very important parameter is the RG of the tip, i.e. the ratio between the diameters of outer glass tube and the inner Pt disc UME. In fact, it makes the expression of CE_{max} clearly dependent on the specific experimental set-up used.

The amount of H_2 evolved hydrogen is directly proportional to FE

$$n_{H_2} = \frac{Q_{cath}}{2F}(F.E.) \qquad (7)$$

where Q_{cath} is the integrated cathodic charge and F the Faraday constant.

In our specific case, a Pt disc UME (radius: 15 μm) embedded in glass (external diameter: 110 μm) was located at a given tip-substrate distance above the center of the substrate electrode in an Ar-saturated phosphate buffer (pH 7.4) and was used to collect the H_2 generated at the substrate electrode. The substrate electrode (generator) was also a UME disc (radius 12.5 μm) onto which films of electrocatalysts were deposited by drop-cast. This ensemble was embedded in a PDMS membrane and tightened at the bottom of a home-made Teflon cell with connecting screws and an O-ring.

Such a procedure does not allow a precise control of the size of the electrocatalyst spot, that in fact largely exceeded the limits of the underlying disc electrode. While a and the RG ratio of the probing tip were easily obtained by optical inspection of the UME, an experimental procedure had to be devised to measure the substrate radius (b) that would take into account the porosity and conductivity of the MWCNTs film and therefore the fact that the effective electroactive surface area (and hence b) might be larger than the underlying disc microelectrode. A first estimation of the electroactive surface area was obtained by comparing the steady state current relative to the oxidation of 1mM ferrocenemethanol (FcMeOH) in 100 mM $LiClO_4$ aqueous solution, measured at the substrate electrode before and after deposition by drop-cast. In Supplementary Fig. 16 the corresponding CVs are shown. The presence of the MWCNTs-based film caused an increase of the stationary currents by more than twenty times, that would

correspond to a ~ 4-fold increase of the "electrode diameter", i.e, an effective radius for the substrate electrode of 50 μm.

This is however just a crude approximation, since the electrical conductivity within the films depends on percolation routes, that are not necessarily isotropically distributed, and the electrode area may in fact assumes much more asymmetric shapes. Since this would affect the maximum tip collection efficiency, the electroactive surface area was therefore independently investigated by SECM, using again the SG/TC mode. The Pt tip (15 μm) was positioned, *via* feedback mode in a 1 mM FcMeOH aqueous solution, at about 25 μm above the sample (Supplementary Fig. 17a).

An image of the electroactive area was thus obtained oxidising a redox probe (FcMeOH) at the substrate (E_{sub}= 0.45 V vs. Ag/AgCl) and, at the same time, scanning the surface with the tip biased at 0 V (vs. Ag/AgCl), so as to detect the continuously generated $FcMeOH^{+}$. Supplementary Fig. 17b shows an image of a representative MWCNTs-based film. As expected, and in line with the results of the above CV investigation, such an area exceeds that of the underlying contact (indicated by the grey dashed circle with radius 12.5 μm), as a consequence of the very good conductivity of MWCNTs. Moreover, the area highlighted by SECM is quite symmetric and may be adequately approximated by an equivalent disc with radius 50 μm (indicated by the blue dashed circle) as estimated by CV.

Finally, in order to determine the faradaic efficiency of the HER process for the electrocatalytic films, the SECM Pt tip (radius: 15 μm) was positioned above the centre of the underlying substrate electroactive area and was approached *via* feedback mode (using FcMeOH 1 mM as a redox mediator) at a z-distance of about 25 μm from the film (corresponding to 50% of the bulk current). The solution was replaced with phosphate buffer, rinsed at least ten times before the following measurement, and the H_2 generated at the substrate was at the end detected at the tip (as schematically shown in Supplementary Fig. 15a).

The current density collected at the tip, as explained above, was affected by two factors: (i) the maximum collection efficiency (CE_{max}), which depends entirely on the geometric characteristics of the SECM detection setup, and (ii) the FE of the hydrogen generation process. CE_{max} was then calculated, at the various distances *d*, using eq. 8, reported in ref. 20.

$$CE_{\max} = 103.38 - 2.1681 \bullet \left(\frac{d}{a/b}\right) + 0.02585 \bullet \left(\frac{d}{a/b}\right)^2 - 0.0001485 \bullet \left(\frac{d}{a/b}\right)^3 + 0.000000322 \bullet \left(\frac{d}{a/b}\right)^4 \quad (8)$$

where the geometric parameters *a* and *b* determined as described above were used. Notice that eq. (8) contains coefficients that were optimised for SECM tips with RG between 3-6, as in our

specific case. In Supplementary Fig. 15b, CE_{max} (black dots and line) and the experimental collection efficiency obtained in the SG/TC experiments (red dots and line) are reported as a function of the characteristic geometric parameter d/(a/b). The comparison of the two functions allowed us to evaluate the F.E. of the HER process. The best fit line that approximates all CE values was obtained multiplying eq. 8 by 0.9. In conclusion, the HER process takes place at the **f-MWCNTs@Pd/TiO_2** film (at -0.6 V vs RHE) with a faradaic efficiency of 90%.

Supplementary References

(1) Verlato, E., Cattarin, S., Comisso, N., Gambirasi, A., Musiani, M., Vázquez-Gómez L., *Electrocatal* **2012**, *3*, 48–58.

(2) Hara, M., Linke, U., Wandlowski, Th., *Electrochim. Acta*, **2007**, 52, 5733.

(3) Grden, M., Łukaszewski, M., Jerkiewicz, G., Czerwinski, A., *Electrochim. Acta*, **2008**, 53, 7583-7598.

(4) MessaoudiI, C., Boudier, T., Oscar, C., Sorzano, S., Marco, S. *BMC Bioinformatics* **2007**, *8*, 288

(5) Pettersen, E. F., Goddard, T. D., Huang, C. C., Couch, G. S., Greenblatt, D. M., Meng, E. C. and Ferrin, T. E. *J. Comput. Chem.*, **2004**, 25, 1605.

(6) Dresp, S., Luo, F., Schmack, R., Kuhl, S., Gliech, M., Strasser, P., *Energy Environ. Sci.,* **2016**, 9, 2020.

(7) Sato, N., in *Electrochemistry at metal and semiconductor electrodes*, Elsevier Science B. V., Amsterdam, **1998**.

(8) Tomkiewicz, M., *J. Electrochem. Soc.,* **1979**, *126*, 1505-1510.

(9) Kavan, L., Grätzel, M., Gilbert, S. E., Klemenz, C., Scheel, H. J., *J. Am. Chem. Soc.*, **1996**, *116*, 6716-6723.

(10) Ye, M., Gong, J., Lai, Y., Lin, C., Lin, Z., *J. Am. Chem. Soc.*, **2012**, *134*, 15720–15723.

(11) Zhang, W-D., Jiang, L-C., Ye, J-S., *J. Phys. Chem. C.*, **2009**, *113*, 16247–16253.

(12) Conway, B., Tilak, B. V., *Electrochimica Acta*, **2002**, *47*, 3571-3594.

(13) Le Goff, A., Artero, V., Jousselme, B., Tran, P. D., Guillet, N., Métayé, R., Fihri, A., Palacin S., Fontecave, M., *Science,* **2009**, *326*, 1384-1387.

(14) Helm, M. L., Stewart, M. P., Bullock, R. M., DuBois M. R., DuBois, D. L., *Science*, **2011**, *333*, 863-866.

(15) Liang, H.-W., Bruller, S., Dong, R., Zhang, J., Feng, X., Mullen, K., *Nat. Commun.*, **2015**, 6:7992.

(16) Merki, D., Fierro, S., Vrubel H., Hu, X., *Chem. Sci.*, **2011**, 2, 1262-1267.

(17) Tran, P. D., Nguyen, M., Pramana, S. S., Bhattacharjee, A., Chiam, S. Y., Fize, J., Field, M. J., Artero, V., Wong, L. H., Loo, J., Barber J., *Energy Environ. Sci.*, **2012**, 5, 8912-8916.

(18) Costentin, C., Drouet, S., Robert, M., Savéant, J-M., *J. Am. Chem. Soc.*, **2012**, *134*, 11235–11242.

(19) Wittstock, G., Burchardt, M., Pust, S. E., Shen Y., Zhao, C., *Angew. Chem. Int. Ed.*, **2007**, *46*, 1584-1617.

(20) Sanchez-Sanchez, M. C., Rodriguez-Lopez J., Bard, A. J. *Anal. Chem.*, **2008**, *80*, 3254-3260.

(21) Bard, A. J., Mirkin, M. V., in *Scanning Electrochemical Microscopy*, Marcel Dekker Inc., New York, **2001**.

(22) Amemiya, S., Bard, A. J., Fan, F. F., Mirkin, M. V., Unwin, P. R. *Annu. Rev. Anal. Chem.*, **2008**, *1*, 95-131.
